# Morphological and molecular data reveal one new species of *Coltricia* and one new species of *Sidera* in Hymenochaetales from South China

**DOI:** 10.3897/mycokeys.127.173104

**Published:** 2026-01-15

**Authors:** Wan-Ying Li, Wen Jing, Qian-Xin Guan, Fang Wu

**Affiliations:** 1 State Key Laboratory of Efficient Production of Forest Resources, School of Ecology and Nature Conservation, Beijing Forestry University, Beijing 100083, China Beijing Forestry University Beijing China

**Keywords:** New taxa, phylogenetic analysis, polypore, taxonomy

## Abstract

Two new poroid species of Hymenochaetales, *Coltricia subpusilla* and *Sidera pini*, are described from Anhui Province and Jiangxi Province based on morphological characters and multimarker phylogenetic analyses using a combined ITS, nLSU, and partial *tef*1 dataset. Phylogenetic results revealed that *C. subpusilla* is closely related to *C. pusilla* and that *S. pini* is related to *S. borealis*. *Coltricia subpusilla* was found on the bark of dead *Pinus* and is recognized by its annual, laterally stipitate, small, flabelliform to subcircular pilei, angular pores (2–3 per mm), and smooth to verrucose basidiospores. *Sidera pini* was found on fallen trunks of *Pinus massoniana* and is characterized by annual, resupinate basidiomata with angular pores (8–12 per mm), a dimitic hyphal system, and allantoid basidiospores. Detailed descriptions and illustrations of the two new species are provided.

## Introduction

The order Hymenochaetales (Agaricomycetes, Basidiomycota) was established by Oberwinkler in 1977, with Hymenochaetaceae Donk as the type family (Frey et al. 1977). It is a large order in Agaricomycetes and is globally distributed, comprising 15 families and 84 genera, of which 19 have an uncertain position at the family level (He et al. 2024; Wang and Zhou 2024). The majority of species within Hymenochaetales are poroid and corticioid, exhibiting high morphological diversity and various trophic strategies, including wood-inhabiting and ectomycorrhizal fungi (Tedersoo et al. 2007; Zhou et al. 2023; Dai et al. 2025; Deng et al. 2025; Liu et al. 2025).

The cosmopolitan genus *Coltricia* Gray, typified by *Coltricia perennis* (L.) Murrill, was established in 1821 and is currently placed in the family Hymenochaetaceae (Wu et al. 2022a). The genus is characterized by predominantly poroid and stipitate basidiomata, a monomitic hyphal system lacking clamp connections, and slightly to distinctly thick-walled, brownish basidiospores (Wu et al. 2022a). Species of *Coltricia* are primarily terricolous and have a saprotrophic lifestyle (Wu et al. 2022a, b; Zhao et al. 2023). However, *C. perennis* was reported to form ectomycorrhizae (Tedersoo et al. 2007); therefore, further studies are needed to confirm the lifestyle of other *Coltricia* species. *Coltriciella* Murrill was established by Murrill (1904) and is similar to *Coltricia* in morphological characteristics, except for having verrucose basidiospores. *Coltriciella* formed a monophyletic clade within *Coltricia* based on phylogenetic analyses; thus, it was treated as a synonym of *Coltricia* and merged into *Coltricia* (Wu et al. 2022a). Recently, *Coltricia* has been widely studied worldwide, and more than 20 species have been described and reported in the last 10 years. Thirteen new *Coltricia* species were discovered in South China, primarily distributed in the Yunnan region (Bian and Dai 2015, 2017, 2020; Bian et al. 2016, 2022; Vasco-Palacios 2016; Susan et al. 2018; Valenzuela et al. 2020; Vlasák et al. 2020; Wu et al. 2022a; Patil et al. 2024; Zhang et al. 2024; Zhou et al. 2024). According to Index Fungorum (https://www.indexfungorum.org; accessed on 5 June 2025), the genus *Coltricia* (including former *Coltriciella* species) has 156 records, and 84 species are currently accepted worldwide (Wu et al. 2022a; Zhang et al. 2024; Zhou et al. 2024).

*Sidera* Miettinen & K.H. Larss. was originally proposed by Miettinen and Larsson (2011) based on molecular analyses and morphological characteristics and is currently placed in the family Sideraceae (He et al. 2024). It is distinguished by resupinate, white to cream or buff basidiomata when fresh, poroid or hydnoid hymenophores, a monomitic or dimitic hyphal system with clamp connections in generative hyphae, loosely arranged skeletal hyphae, the presence of rosette-like crystals, and allantoid to lunate, acyanophilous basidiospores that are negative in Melzer’s reagent (Miettinen and Larsson 2011; Liu et al. 2022, 2023). Species of *Sidera* mainly grow on rotting wood and cause white rot (Liu et al. 2021, 2023). In recent years, the species diversity of *Sidera* has been extensively studied in China, North America, and Europe. Five new species were discovered in South China, with the majority reported from Hainan Province and Tibet (Dai 2010; Du et al. 2019, 2020; Liu et al. 2021, 2022, 2023; Xu et al. 2023; Fryssouli et al. 2024). According to Index Fungorum (https://www.indexfungorum.org; accessed on 5 June 2025), 21 names of *Sidera* are recorded, and 19 species are currently accepted (Xu et al. 2023; Fryssouli et al. 2024).

During our investigations of wood-rotting fungi in South China, four poroid specimens were collected and identified as belonging to two new species of Hymenochaetales, *Coltricia subpusilla* sp. nov. and *Sidera pini* sp. nov., based on morphological characteristics and molecular data from ITS, nLSU, and *tef*1 sequences. This study enriches the species diversity of Hymenochaetales in South China.

## Materials and methods

### Morphological studies

Voucher specimens are deposited at the Fungarium of Beijing Forestry University (**BJFC**). Macro-morphological descriptions were based on field notes and laboratory observations. Microscopic measurements and drawings were made from slide preparations of dried tissues stained with Cotton Blue and Melzer’s reagent. The following abbreviations were used in the descriptions: IKI = Melzer’s reagent; IKI– = negative in Melzer’s reagent; KOH = 5% potassium hydroxide; CB = cotton blue; CB– = acyanophilous; CB+ = cyanophilous; L = mean spore length (arithmetic average of all spores); W = mean spore width (arithmetic average of all spores); Q = L/W ratio for each specimen studied; n (a/b) = number of spores (a) measured from a given number (b) of specimens. Special color terms followed Petersen (1996) and Anonymous (1969).

### DNA extraction and sequencing

A CTAB rapid plant genome extraction kit-DNA (Aidlab Biotechnologies Co., Ltd) was used to extract total genomic DNA from dried specimens of the new collections according to the manufacturer’s instructions, with some modifications (Wu et al. 2022a). The primer pair ITS4 and ITS5 was used for amplification of the ITS region, whereas the primer pair LR0R and LR7 (https://www.biology.duke.edu/fungi/mycolab/primers.htm) was used for amplification of the nuclear large subunit ribosomal DNA (nLSU), and EF1-983F and EF1-1567R were used for *tef*1 (White et al. 1990; Rehner 2001). The PCR procedure for ITS and *tef*1 was as follows: initial denaturation at 95 °C for 3 min, followed by 35 cycles at 94 °C for 40 s, 54 °C for 45 s, and 72 °C for 1 min, with a final extension at 72 °C for 10 min. The PCR procedure for nLSU was as follows: initial denaturation at 94 °C for 1 min, followed by 35 cycles at 94 °C for 30 s, 50 °C for 1 min, and 72 °C for 1.5 min, with a final extension at 72 °C for 10 min. The PCR products were purified and sequenced at the Beijing Genomics Institute, China, using the same primers. All newly generated sequences were submitted to GenBank and are listed in Table 1.

**Table 1.** Taxon information and sequences used in this study.

Species	Specimen No.	Country	GenBank accession no.		
			ITS	nLSU	TEF1
*Coltricia abieticola*	Cui 12276	China	KU360673	KU360643	KY693912
*C. abieticola*	Cui 12312	China	KU360674	KU360644	–
*C. australica*	TU 103694^T^	Australia	–	AM412243	–
*C. austrosinensis*	Dai 13093^T^	China	KU360670	KU360640	KY693913
*C. austrosinensis*	Dai 13098	China	KU360671	KU360640	–
*C. barbata*	AMV 1866	Colombia	KT724137	–	–
*C. barbata*	AMV 1925	Colombia	KT724136	KT724149	–
*C. baoshanensis*	Cui 8147	China	KX364799	KX364819	–
*C. baoshanensis*	Dai 13075^T^	China	KX364800	KX364820	KY693953
*C. cinnamomea*	Cui 12549	China	KY693728	KY693742	KY693916
*C. cinnamomea*	Cui 12584	China	KY693729	KY693743	KY693917
*C. cinnamomea*	TN 8199	Finland	–	MF318906	–
*C. confluens*	TAA 181460	Estonia	AM412241	AM412241	–
*C. confluens*	TF 072287	USA	MN121008	MN121008	–
*C. crassa*	Cui 10255	China	KU360678	KU360647	KY693921
*C. crassa*	Dai 15163	China	KU360679	KU360648	KY693922
*C. dependens*	Dai 10944	China	KY693737	KY693757	–
*C. dependens*	Cui 9210	China	KY693738	KY693758	–
*C. fimbriata*	Dai 22300^T^	China	OL691607	OL691616	–
*C. fragilissima*	Dai 16636	Thailand	KY693733	KY693749	–
*C. focicola*	Dai 16090	China	KX364786	KX364805	KY693923
*C. focicola*	Dai 26383	China	OR964386	OR964380	–
*C. globosa*	Cui 7545^T^	China	KJ540930	KJ000226	KY693954
*C. globosa*	Dai 18420	Vietnam	MT174245	MT174238	–
*C. hamata*	AMV 1897	Colombia	KT724146	KT724150	–
*C. hamata*	AMV 2076	Colombia	KT724142	KT724151	–
*C. hirtipes*	Dai 16647	Thailand	KY693734	KY693750	–
*C. hirtipes*	Dai 16651	Thailand	–	KY693751	–
*C. kinabaluensis*	Dai 13957	Thailand	KX364787	KX364806	KY693924
*C. kinabaluensis*	Dai 13958	Thailand	KX364788	KX364807	KY693925
*C. lateralis*	Cui 12563^T^	China	KX364789	KX364808	KY693926
*C. lateralis*	Dai 13564	China	KX364790	KX364809	KY693927
*C. lenis*	Dai 22367	China	OL691608	OL691617	–
*C. lenis*	Dai 22374^T^	China	OL691609	OL691619	–
*C. macropora*	Cui 9019^T^	China	KU360680	KJ000220	–
*C. macropora*	Cui 9039	China	KU360681	KJ000221	KY693928
*C. minima*	Dai 15206^T^	China	KU360682	KU360649	KY693929
*C. minima*	Dai 15222	China	KU360683	KU360650	KY693930
*C. minor*	Dai 16088	China	KU360684	KU360651	KY693931
*C. minuscula*	BO22806^T^	Indonesia	KX086684	–	–
*C. montagnei*	Cui 10169	China	KU360685	KU360652	KY693932
*C. montagnei*	Dai 12137	China	–	KX364810	KY693933
*C. montagnei*	MF 96-96	USA	–	AY039683	–
*C. navispora*	MCA 3921	Guyana	KC155387	KC155386	–
*C. navispora*	TH 9529	Guyana	KT339262	–	–
*C. oblectabilis*	AMV 2255	Colombia	KT354690	–	–
*C. oblectabilis*	TH 9187	Guyana	KC155387	KC155387	–
*C. perennis*	Cui 10318	China	KU360686	KJ000224	KY693934
*C. perennis*	Cui 10319	China	KU360687	KU360653	KY693935
*C. perennis*	JV 0809/66	USA	KX364791	KX364811	–
*C. pseudodependens*	Cui 8138^T^	China	KJ540931	KJ000227	–
*C. pseudodependens*	Cui 12582	China	KX364801	KX364821	KY693955
*C. pusilla*	Dai 15168	China	KU360701	KU360667	KY693956
*C. pusilla*	Dai 26381	China	OR964387	OR964381	–
*C. pusilla*	MN 26.7.95	Japan	–	AY059060	–
*C. pyrophila*	Cui 10314	China	KU360689	KU360655	KY693937
*C. pyrophila*	Cui 10411	China	KU360690	KU360656	KY693938
*C. pyrophila*	Cui 12553	China	KX364792	KX364812	KY693939
*C. raigadensis*	AMH 10511^T^	India	OR072877	–	–
*C. raigadensis*	MMH 1211	India	OR072932	OR053821	–
*C. rigida*	Dai 13622a^T^	China	KX364793	KX364813	–
*C. rigida*	Dai 16322	China	KX364794	KX364814	KY693941
*C. sinoperennis*s	Dai 11625	China	KY693735	KY693753	
*C. sinoperennis*	Dai 13095	China	KY693736	KY693754	–
*C. sonorensis*	RV 13144^T^	Mexico	–	HQ439179	–
*C. strigosipes*	Dai 15145	China	KX364795	KX364815	KY693942
*C. strigosipes*	Dai 15586	China	KU360692	KU360658	KY693943
*C. subcinnamomea*	Dai 17016^T^	China	KY693740	KY693755	–
*C. subcinnamomea*	Dai 17022	China	–	KY693756	–
*C. subglobosa*	Dai 25569	China	OR964388	OR964382	–
*C. subglobosa*	Yuan 6253	China	–	KX364822	–
***C. subpusilla***	**Wu 2076^T^**	**China**	PV919824*	PV919828*	PV928713*
***C. subpusilla***	**Wu 2077**	**China**	PV919825*	PV919829*	PV928714*
*C. subverrucata*	Dai 12919	China	MT174242	MT174235	MT133895
*C. subverrucata*	Dai 15600^T^	China	MT174243	MT174236	MT133896
*C. tenuihypha*	Dai 22684^T^	China	OL691610	OL691620	–
*C. tenuihypha*	Dai 22690	China	OL691611	OL691621	–
*C. tibetica*	Cui 12208^T^	China	MZ484551	MZ437407	–
*C. velutina*	Dai 16980	China	–	KY693752	–
*C. verrucata*	Dai 15120	China	KU360694	KU360660	KY693945
*C. verrucata*	Dai 15125	China	KU360695	KU360661	KY693946
*C. weii*	Cui 12624	China	KX364796	KX364816	KY693950
*C. weii*	Dai 13422	China	KX364797	KX364817	KY693951
*C. weii*	Dai 25824	China	OR964389	OR964383	–
*C. wenshanensis*	Dai 15585^T^	China	KX364798	KX364818	KY693952
*C. wuyiensis*	Dai 25601	China	OR964390	OR964384	–
*C. wuyiensis*	Dai 26431^T^	China	OR964391	OR964385	–
*C. yunnanensis*	CLZhao 4204^T^	China	OR668921	OR708662	–
*C. zixishanensis*	CLZhao 7706^T^	China	OR668922	OR708662	–
*Fomitiporella chinensis*	Cui 11230	China	KX181309	KY693759	KY693958
*Inonotus griseus*	Dai 13436	China	KX364802	KX364823	KY693959
*Sidera americana*	Dai 19173	Canada	MW198477	MW192005	–
*S. americana*	Dai 12730^T^	USA	MW198478	–	–
*S. borealis*	Dai 22822	China	OM974254	OM974246	–
*S. borealis*	Cui 11216^T^	China	MW198485	–	–
*S. borealis*	Dai 23962	China	OQ134534	–	–
*S. borealis*	Dai 23803	China	OQ134535	–	–
*S. borealis*	Dai 24120	China	OQ134533	–	–
*S. borealis*	Dai 24187	China	OQ134536	OQ134528	–
*S. borealis*	Dai 23960	China	OQ134537	–	–
*S. inflata*	Cui 13610^T^	China	MW198480	–	–
*S. lenis*	Dai 22834	China	OQ134538	OQ134529	–
*S. lenis*	Dai 22854	China	OQ134539	OQ134530	–
*S. lenis*	Miettinen 11036	Finland	FN907914	FN907914	–
*S. lowei*	Miettinen X419	Venezuela	FN907917	FN907917	–
*S. lowei*	Dollinger 922	USA	KY264044	–	–
*S. lowei*	Miettinen X426	New Zealand	FN907919	FN907919	–
*S. lunata*	JS 15063	Norway	DQ873593	DQ873593	–
*S. malaysiana*	Dai 18570^T^	Malaysia	MW198481	MW192007	–
*S. minutipora*	Gates FF257	Australia	FN907922	FN907922	–
*S. minutipora*	Cui 16720	Australia	MN621349	MN621348	–
*S. minutissima*	Dai 19529^T^	Sri Lanka	MN621352	MN621350	–
*S. minutissima*	Dai 22495	China	OM974248	OM974240	–
*S. minutissima*	Dai 18471A	China	MW198482	MW192008	–
*S. parallela*	Dai 22038	China	MW477793	MW474964	–
*S. parallela*	Cui 10346^T^	China	MK346145	–	–
*S. parallela*	Cui 10361	China	MK346144	–	–
*S. parallela*	Dai 22635	China	OQ134540	OQ134531	–
***S. pini***	**Wu 1847**	**China**	PV919826*	PV919830*	–
***S. pini***	**Wu 1848^T^**	**China**	PV919827*	PV919831*	–
*S. punctata*	Dai 22119^T^	China	MW418438	MW418437	–
*S. roseobubalina*	Dai 11277^T^	China	MW198483	–	–
*S. salmonea*	Dai 23343	China	OM974249	OM974241	–
*S. salmonea*	Dai 23354	China	OM974250	OM974242	–
*S. salmonea*	Dai 23428	China	OM974251	OM974243	–
*S. salmonea*	Dai 23612^T^	China	–	OM974247	–
*S. srilankensis*	Dai 19581	Sri Lanka	MN621345	MN621347	–
*S. srilankensis*	Dai 19654^T^	Sri Lanka	MN621344	MN621346	–
*S. tenuis*	Dai 18697^T^	Singapore	MK331865	MK331867	–
*S. tenuis*	Dai 18698	Singapore	MK331866	MK331868	–
*S. tianshanensis*	Cui 19143^T^	China	OP920995	OP920987	–
*S. tianshanensis*	Cui 19132	China	OP920994	OP920986	–
*S. tibetica*	Dai 23407	China	OM974252	OM974244	–
*S. tibetica*	Dai 23648^T^	China	OM974253	OM974245	–
*S. tibetica*	Dai 21057	Belarus	MW198484	MW192009	–
*S. tibetica*	Dai 22151	China	MW477794	MW474965	–
*S. vesiculosa*	BJFC025367	Singapore	MH636565	MH636567	–
*S. vesiculosa*	Dai 17845^T^	Singapore	MH636564	MH636566	–
*S. vulgaris*	HUBO 7745	Italy	PP275217	PP275227	–
*S. vulgaris*	HUBO 8296	Italy	PP275218	PP275228	–
*S. vulgaris*	SALA Fungi 3749	Spain	PP275220	–	–
*S. vulgaris*	SALA Fungi 4105	Spain	PP275222	–	–
*S. vulgaris*	Ryvarden 37198	New Zealand	FN907918	FN907918	–
*Skvortzovia furfuracea*	KHL 11738	Finland	DQ873648	DQ873648	–
*Sk. furfurella*	KHL 10180	Puerto Rico	DQ873649	DQ873649	–

New species are shown in bold. * Newly generated sequences for this study. ^T^ represents type specimens.

### Phylogenetic analysis

New sequences generated in this study and reference sequences retrieved from GenBank (Table 1) were partitioned into ITS1, 5.8S, ITS2, nLSU, and *tef*1 and then aligned separately using MAFFT v.7.526 (Katoh et al. 2019; http://mafft.cbrc.jp/alignment/server/) with the G-INS-I iterative refinement algorithm and optimized manually in BioEdit v.7.0.5.3 (Hall 1999). The separate alignments were then concatenated using PhyloSuite v.1.2.2 (Zhang et al. 2020). Phylogenetic trees of *Coltricia* and *Sidera* were constructed using the concatenated ITS1+5.8S+ITS2+nLSU+*tef*1 dataset and the concatenated ITS1+5.8S+ITS2+nLSU dataset, respectively, and phylogenetic analyses were performed using maximum likelihood (ML) and Bayesian inference (BI). The final alignments and the resulting topologies were deposited in TreeBASE (http://www.treebase.org) under accessions 32218 and 32242.

RAxML v.7.2.8 was used to infer ML trees under the GTR+I+G model of site substitution, including estimation of gamma-distributed rate heterogeneity and a proportion of invariant sites (Stamatakis 2006). Branch support was evaluated using a bootstrap method with 1,000 replicates (Hillis and Bull 1993). BI was performed using MrBayes 3.2.7 with the best-fit partitioning scheme and substitution model determined by ModelFinder v2.2.0 (Ronquist et al. 2012; Kalyaanamoorthy et al. 2017). Four Markov chains were run for two independent runs from random starting trees for one million generations in the phylogenetic analyses of *Coltricia* and *Sidera* until the split deviation frequency value reached < 0.01, and trees were sampled every 1,000 generations. The first 25% of the sampled trees were discarded as burn-in, and the remaining trees were used to reconstruct a majority-rule consensus tree and to calculate Bayesian posterior probabilities (BPP) for the clades. The phylogenetic trees were visualized in FigTree v.1.4.4 (Rambaut 2018). Branches receiving ML bootstrap support (BS) and BPP values ≥ 75% and ≥ 0.90, respectively, were considered to be significantly supported.

## Results

### Phylogenetic analyses

**Figure 1.**
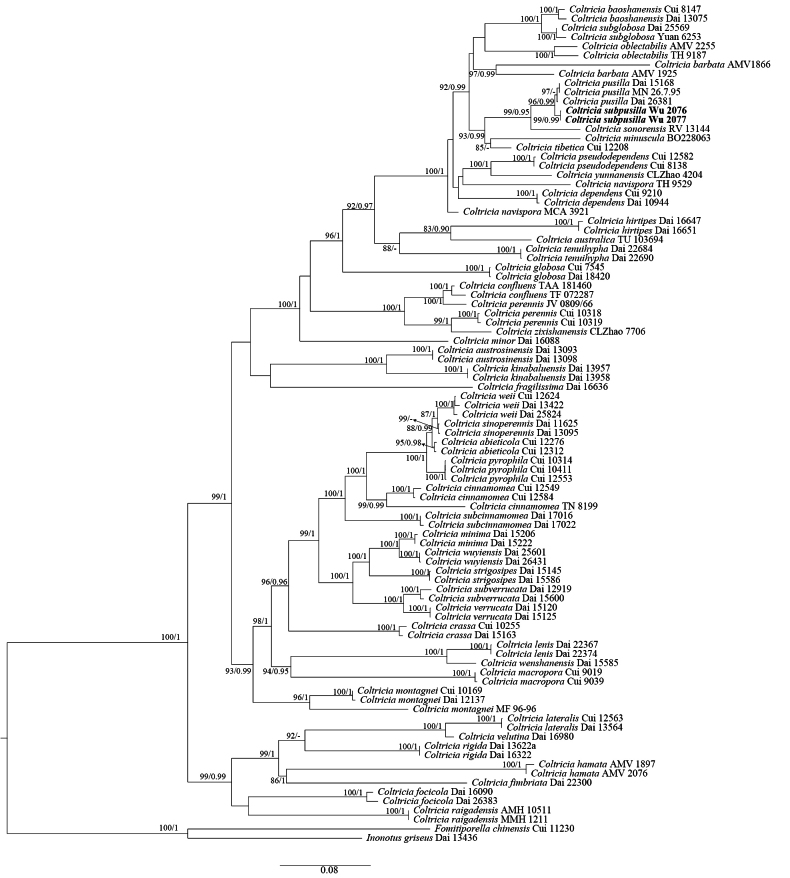
Maximum likelihood (ML) phylogenetic tree illustrating the phylogeny of *Coltricia* based on the combined ITS+nLSU+*tef*1 dataset. Branches are labeled with ML bootstrap values (BS) greater than 75% and Bayesian posterior probabilities greater than 0.90. The new species is shown in bold.

In the phylogenetic analysis of *Coltricia*, the combined ITS+nLSU+*tef*1 dataset included sequences from 91 fungal collections representing 49 taxa. The final alignment comprised a total of 3,318 nucleotide positions, including 747 bases of ITS1, 158 bases of 5.8S, 422 bases of ITS2, 1,411 bases of nLSU, and 580 bases of *tef*1. The nLSU region was relatively conserved, whereas ITS and *tef*1 showed higher variability, potentially providing more phylogenetic information. *Fomitiporella chinensis* (Pilát) Y.C. Dai, X.H. Ji & Vlasák and *Inonotus griseus* L.W. Zhou were used as outgroups following Bian et al. (2022). ModelFinder proposed the models HKY+F+G4 for ITS1, K2P+G4 for 5.8S, GTR+F+G4 for ITS2, GTR+F+I+G4 for nLSU, and SYM+I+G4 for *tef*1 for the Bayesian analysis. The Bayesian inference (BI) analysis resulted in an average standard deviation of split frequencies of 0.009848. The maximum likelihood (ML) and BI trees were similar in topology; therefore, only the ML topology is presented, with branch support values from ML (≥75%) and Bayesian posterior probabilities (BPP ≥0.90) shown (Fig. 1). The phylogeny inferred from the ITS+nLSU+*tef*1 sequences (Fig. 1) showed that our two specimens – representing the new species *Coltricia subpusilla* – formed a well-supported sister lineage (99/0.99) to *C. pusilla* Imazeki & Kobayasi. There are approximately 2.2% differences in the ITS sequences between *C. subpusilla* and *C. pusilla*.

**Figure 2.**
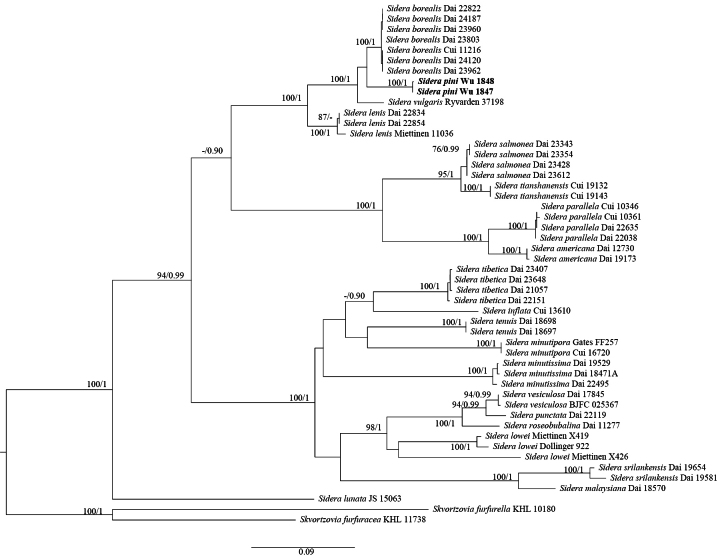
Maximum likelihood (ML) phylogenetic tree illustrating the phylogeny of *Sidera* based on the combined ITS+nLSU dataset. Branches are labeled with ML bootstrap values (BS) greater than 75% and Bayesian posterior probabilities greater than 0.90. The new species is shown in bold.

In the phylogenetic analysis of *Sidera*, the combined ITS+nLSU dataset included sequences from 54 fungal specimens representing 22 taxa. The final alignment comprised a total of 2,186 nucleotide positions, including 347 bases of ITS1, 129 bases of 5.8S, 350 bases of ITS2, and 1,360 bases of nLSU. The nLSU region was relatively conserved, whereas the ITS region was more variable. *Skvortzovia furfuracea* (Bres.) G. Gruhn & Hallenb. and *Sk. furfurella* (Bres.) Bononi & Hjortstam were used as outgroups following Fryssouli et al. (2024). ModelFinder proposed the models HKY+F+G4 for ITS1, GTR+F+I+G4 for 5.8S, HKY+F+G4 for ITS2, and GTR+F+I for nLSU for the Bayesian analysis. The BI analysis resulted in an average standard deviation of split frequencies of 0.009951. Because the ML and BI trees showed similar topologies, only the ML topology is presented, with statistical support values from ML (≥75%) and BPP (≥0.90) shown (Fig. 2). The phylogeny inferred from the combined ITS+nLSU sequences indicated that our two specimens – representing the new species *Sidera pini* – formed a distinct lineage with high support (100/1.00) and were closely related to *S. borealis* Z.B. Liu & Yuan Yuan and *S. vulgaris* (Fr.) Miettinen. There are more than 9% differences between the ITS sequences of *S. pini* and *S. borealis*.

### Taxonomy

#### Coltricia subpusilla

Taxon classificationFungiHymenochaetalesHymenochaetaceae

F. Wu, W.Y. Li, W. Jing & Y.C. Dai, sp. nov.

C27B2385-B647-5EC7-9092-1B3887CBDBDA

860043

Figs 3, 4

##### Diagnosis.

*Coltricia subpusilla* is distinguished by its annual, laterally stipitate, small, flabelliform to subcircular pilei, angular pores (2–3 per mm), and basidiospores that are smooth to verrucose, usually with one guttule and CB+.

**Figure 3.**
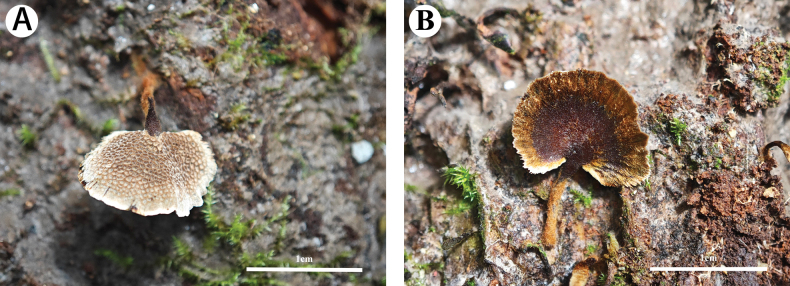
Basidiomata of *Coltricia subpusilla* (holotype, Wu 2076). **A**. Pore surface; **B**. Pileal surface.

**Figure 4.**
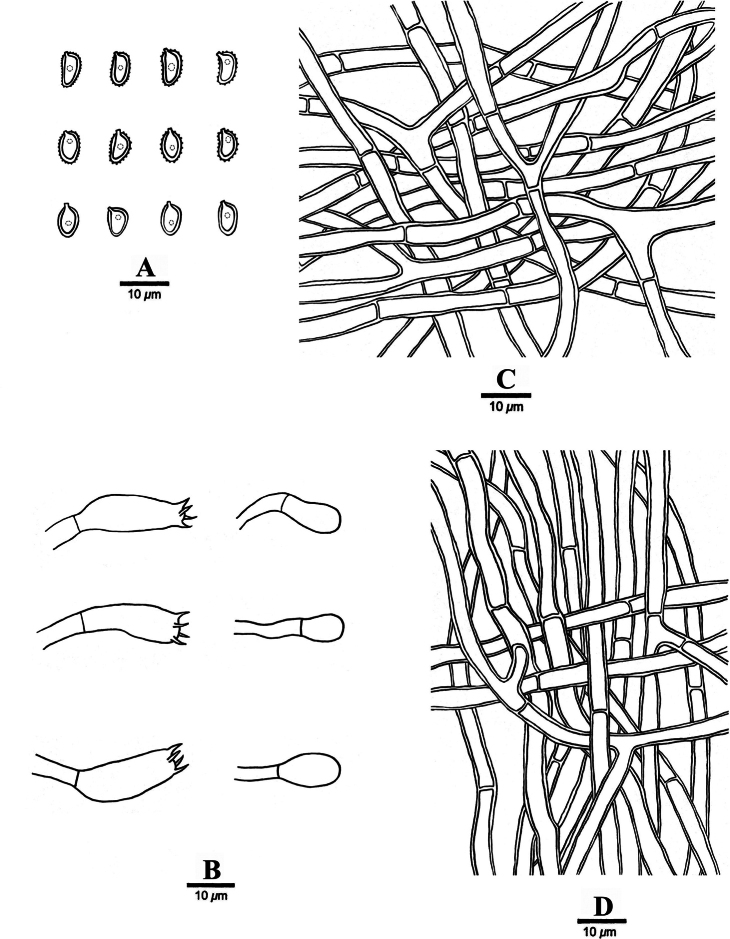
Microscopic structures of *Coltricia subpusilla* (holotype, Wu 2076). **A**. Basidiospores; **B**. Basidia and basidioles; **C**. Hyphae from context; **D**. Hyphae from trama.

##### Holotype.

China. • Anhui Province, Anqing, Yuexi County, Laibang Town, 30°54'32"N, 116°14'7"E, 725 m asl., 4 July 2024, on bark of dead *Pinus*, F. Wu leg., Wu 2076 (BJFC 046384, holotype).

##### Etymology.

*Subpusilla* (Lat.): Referring to its macro-morphological resemblance to *Coltricia pusilla*.

##### Description.

***Basidiomata***. Annual, laterally stipitate, solitary to gregarious, soft fibrous without odor or taste when fresh, becoming soft corky when dry. Pilei small, flabelliform to more or less circular, flat to slightly depressed towards the stipitate, up to 13 mm in diam and 1 mm thick at center. Pileal surface velutinate, radially aligned fine hair extending to the margin, reddish brown to honey yellow from center to margin, slightly shiny, margin thinning out and lobed. Pore surface fawn color to greyish brown when fresh, become greyish brown when dry; pores angular, 2–3 per mm; dissepiments thin, entire. Context greyish brown to clay-buff color when dry, soft corky, up to 0.5 mm thick. Tubes fawn color, distinctly deeper than context in color, soft corky, up to 0.5 mm long. Stipe reddish brown, corky and finely velutinate when fresh, up to 0.9 cm long and 1 mm in diam, with a more or less swollen tip.

***Hyphal structure***. Hyphal system monomitic; generative hyphae simple septate, tissue darkening but otherwise unchanged in KOH.

***Context***. Contextual hyphae buff color to cinnamon-buff color, thick-walled with a wide lumen, rarely branched, frequently simple septate, straight, more or less regularly arranged, 4.0–9.4 μm in diam; hyphae in stipe clay-buff color, thick-walled with a narrow lumen, rarely septate and branched, sometimes sclerified, distinctly narrower than those in context, loosely interwoven, 3.7–5.5 μm in diam.

***Tubes***. Tramal hyphae buff yellow color to cinnamon-buff color, slightly thick-walled with a wide lumen, rarely branched, frequently simple septate, more or less flexuous, loosely interwoven, 3–5 μm in diam. Cystidia and cystidioles absent. Basidia clavate, with four sterigmata and a simple septum at the base, 20–25 × 6–10 μm; basidioles similar in shape but slightly smaller.

***Basidiospores***. Navicular to ellipsoid, cinnamon-buff color, thick-walled, smooth to verrucose, usually with one guttule, IKI–, CB+, (4.8–)5.9–7.4(–9.0) × (2.9–)3.4–4.5(–5.1) μm, L = 6.70 μm, W = 3.95 μm, Q = 1.65–1.74 (n = 60/2).

##### Additional specimen examined (paratype).

China. • Anhui Province, Anqing, Yuexi County, Laibang Town, 30°54'32"N, 116°14'7"E, 722 m asl., 4 July 2024, on dead bark of *Pinus*, F. Wu leg., Wu 2077 (BJFC 046385).

#### Sidera pini

Taxon classificationFungiHymenochaetalesRepetobasidiaceae

F. Wu, W.Y. Li, W. Jing & Y.C. Dai, sp. nov.

679E9258-B285-5465-B2E5-E6CA1E6A7419

860044

Figs 5, 6

##### Diagnosis.

*Sidera pini* can be diagnosed by annual, resupinate basidiomata with angular pores (8–12 per mm), dimitic hyphal system, presence of cystidioles (cystidia absent), and allantoid basidiospores that occasionally with one or two guttules.

**Figure 5.**
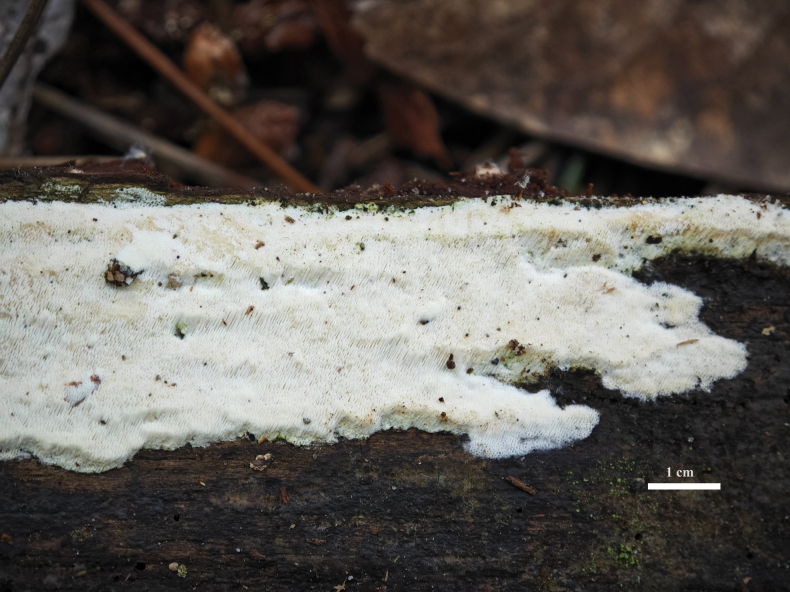
Basidiomata of *Sidera pini* (holotype, Wu 1848).

**Figure 6.**
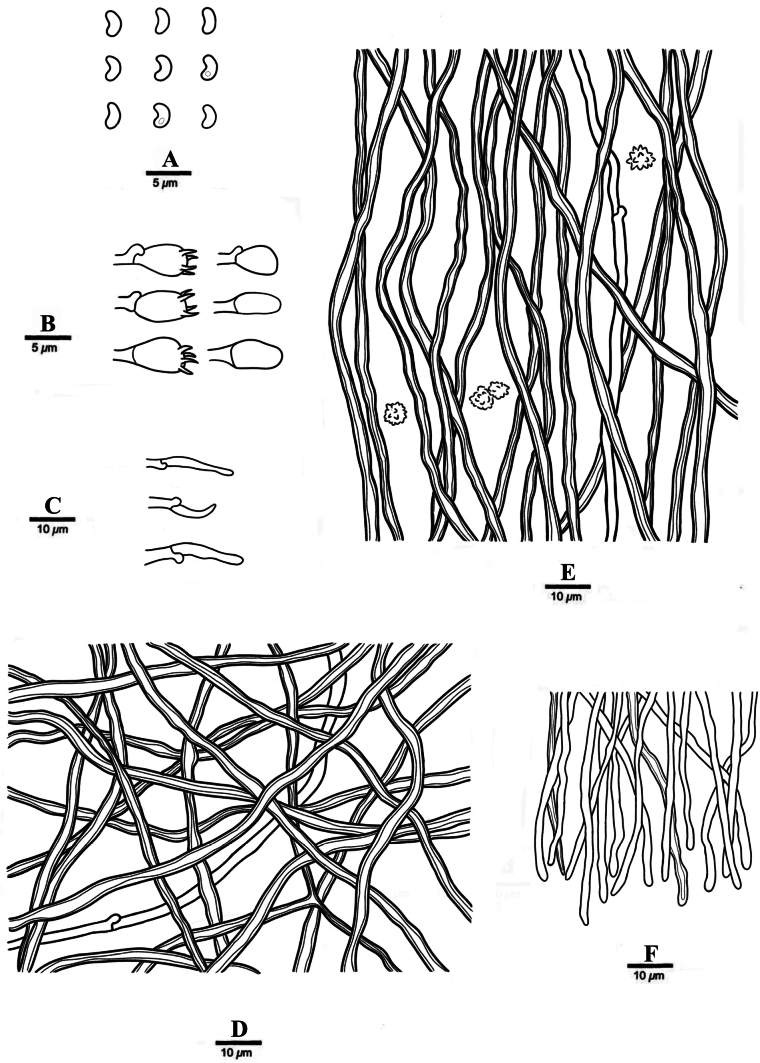
Microscopic structures of *Sidera pini* (holotype, Wu 1848). **A**. Basidiospores; **B**. Basidia and basidioles; **C**. Cystidioles; **D**. Hyphae from subiculum; **E**. Hyphae from trama; **F**. Hyphae at dissepiment edge.

##### Holotype.

China. • Jiangxi Province, Jian, Jinggangshan County, Jinggangshan, 26°32'22"N, 114°8'54"E, 985 m asl., 12 July 2024, on fallen trunk of *Pinus massoniana*, F. Wu leg., Wu 1848 (BJFC 046157, holotype).

##### Etymology.

*Pini* (Lat.): Referring to the species growth on *Pinus* sp.

##### Description.

***Basidiomata***. Annual, resupinate, soft and white when fresh, soft corky when dry, up to 12 cm long, 2.1 cm wide, and 2.5 mm thick at center. Pore surface white to cream color when fresh, becoming cream color to buff color when dry; pores angular, 8–12 per mm; dissepiments thin, lacerate. Margin fertile, not differentiate. Subiculum very thin to almost absent; tubes concolorous with pore surface, up to 2 mm long.

***Hyphal structure***. Hyphal system dimitic; generative hyphae with clamp connections; skeletal hyphae dominant; all hyphae IKI–, CB–; tissue unchanged in KOH.

***Subiculum***. Generative hyphae hyaline, thin-walled, unbranched, 1–2 μm in diam; skeletal hyphae dominant, thick-walled with a narrow to medium lumen, occasionally branched, flexuous, interwoven, 2–3 μm diam.

***Tubes***. Generative hyphae hyaline, thin-walled, unbranched, 1–2 μm in diam, dominating at dissepiment edges; skeletal hyphae dominant in trama except dissepiment edges, thick-walled with a narrow to medium lumen, occasionally branched, flexuous, interwoven, 2–3 μm in diam. Rosette-like crystals abundant, 3–11 μm in diam. Cystidia absent; cystidioles present, fusoid, hyaline, thin-walled, basally slightly swollen, with an obtuse or capitate tip and often hyphoid neck, 9.0–11.2 × 2.8–3.6 μm. Basidia barrel-shaped, hyaline, with four sterigmata and a basal clamp connection, 6.2–7.0 × 3.2–4.2 μm; basidioles in shape similar to basidia, but slightly shorter.

***Basidiospores***. Allantoid, hyaline, thin-walled, smooth, occasionally with one or two guttules, IKI–, CB–, (1.7–)2.2–2.9(–3.8) × 1.1–1.5(–1.6) μm, L = 2.63 μm, W = 1.32 μm, Q = 1.98–1.99 (n = 60/2).

##### Additional specimen examined.

China. • Jiangxi Province, Jian, Jinggangshan County, Jinggangshan, 26°32'22"N, 114°8'54"E, 985 m asl., 12 July 2024, on fallen trunk of *Pinus massoniana*, F. Wu leg., Wu 1847 (BJFC 046156).

## Discussion

In this study, two new species of Hymenochaetales – *Coltricia subpusilla* and *Sidera pini* – are described from South China based on morphological characters and phylogenetic analyses.

*Coltricia subpusilla* is characterized by its small (about 1 cm in diam), laterally stipitate basidiomata; a reddish brown to honey yellow pileal surface from the center to the margin; a fawn-colored to grayish brown pore surface when fresh; pores 2–3 per mm; and navicular to ellipsoid basidiospores. Phylogenetically, *C. subpusilla* forms a sister lineage to *C. pusilla*, and both are closely related to *C. minuscula* (Susan, Retn. & Sukarno) Y.C. Dai & F. Wu, *C. sonorensis* (R. Valenz., Esqueda & Decock) Y.C. Dai & F. Wu, and *C. tibetica* Y.C. Dai & F. Wu (Fig. 1). Morphologically, *C. minuscula* and *C. subpusilla* have similar pores (2–3 per mm) and basidiospore sizes (5.8–7.2 × 3.8–4.8 µm vs. 5.9–7.4 × 3.4–4.5 µm), but the former species has pendent and smaller basidiomata (up to 4 mm vs. up to 13 mm in diam), basidia with two sterigmata, and finely verruculose basidiospores (Susan et al. 2018). *Coltricia pusilla* differs from *C. subpusilla* by its coriaceous, glabrous, and smaller pilei (2–10 × 2–7 mm wide vs. up to 13 mm in diam), regular pores, entirely verrucose basidiospores, smaller basidia (6.5–7.5 × 4.5–5.0 µm vs. 20.0–25.0 × 6.0–10.0 µm), and larger basidiospores (8.2–9.8 × 5.0–5.5 µm vs. 5.9–7.4 × 3.4–4.5 µm; Núñez and Ryvarden 2000). *Coltricia sonorensis* differs from *C. subpusilla* by its entirely verrucose and larger basidiospores (8.0–10.5 × 4.0–5.0 µm vs. 5.9–7.4 × 3.4–4.5 µm), growth on soil, and distribution in Mexico (Valenzuela et al. 2011). *Coltricia tibetica* and *C. subpusilla* appear to prefer growth on dead wood and are distributed in China, but the former species has larger basidiomata (up to 3.0 cm vs. up to 1.3 cm in diam), a longer stipe (up to 2.0 cm vs. 0.9 cm long), and produces acyanophilous and significantly larger basidiospores (8.2–9.8 × 5.0–5.5 µm vs. 5.9–7.4 × 3.4–4.5 µm; Wu et al. 2022a).

*Sidera pini* is characterized by annual, resupinate basidiomata; a white to cream pore surface when fresh that becomes cream to buff when dry; pores 8–12 per mm; a dimitic hyphal system; and allantoid basidiospores. Morphologically, *S. pini* resembles *S. malaysiana* Z.B. Liu & Y.C. Dai by having similar pores (8–12 per mm vs. 9–11 per mm) and basidiospore widths (1.1–1.5 µm vs. 1.0–1.2 µm), but *S. malaysiana* has longer basidiospores (2.9–3.2 µm vs. 2.2–2.9 µm long), distinctly larger basidia (7.8–15 × 3.0–4.3 µm vs. 6.2–7.0 × 3.2–4.2 µm), and is distributed in Malaysia (Liu et al. 2021). Phylogenetically, *S. pini* is closely related to *S. borealis* and *S. vulgaris* (Fig. 2), but *S. borealis* differs from *S. pini* by its longer and narrower basidiospores (3.9–4.1 × 1.0–1.1 µm vs. 2.2–2.9 × 1.1–1.5 µm) and growth on fallen angiosperm trunks (Liu et al. 2023). *Sidera vulgaris* (Fr.) Miettinen differs by having larger pores (6–7 per mm vs. 8–12 per mm) and larger basidiospores (2.9–3.6 × 0.9–1.4 µm vs. 2.2–2.9 × 1.1–1.5 µm; Niemelä and Dai 1997).

Although one new species each is described from *Coltricia* and *Sidera*, the delimitation of some previously described species remains unresolved because classical morphological species were often not accurately identified during early sequencing efforts 20 years ago. As a result, GenBank contains multiple distinct and information-wise divergent sequences of, for example, *C. cinnamomea* (Jacq.) Murrill, *C. perennis*, *C. montagnei* (Fr.) Murrill, *C. focicola* (Berk. & M.A. Curtis) Murrill, and *S. lenis* (P. Karst.) Miettinen. For instance, two highly divergent accessions of *C. cinnamomea* appear in the phylogeny in Fig. 1 – one from China and one from Finland – and the sequences of *S. lenis* from China and Finland also differ substantially, sharing only 97% similarity in the ITS region. Because reference sequences from type material are lacking, it remains unclear which, if any, of these sequences represent the species in a strict sense. However, these species were originally described from outside China and are distantly related to the two new species described here. We therefore consider intercontinental distributions to be highly improbable for these two genera, and the divergent sequences from different continents likely represent distinct species. To resolve these long-standing taxonomic issues, additional specimens from type localities will be required for comprehensive morphological and molecular re-examinations.

Wood-inhabiting fungi play key roles in material circulation and energy flow in forest ecosystems. Hymenochaetales is a large and extensively studied order of wood-inhabiting fungi that exhibits substantial morphological and genetic diversity (Susan et al. 2018; Xu et al. 2023; Fryssouli et al. 2024; Wu et al. 2022a; Liu et al. 2021, 2022, 2023, 2025). Numerous new species of *Coltricia* and *Sidera* have been discovered in China (Liu et al. 2021, 2022, 2023; Bian et al. 2022; Wu et al. 2022a). The description of *C. subpusilla* and *S. pini* further improves our understanding of the species diversity of Hymenochaetales in China.

## Supplementary Material

XML Treatment for Coltricia subpusilla

XML Treatment for Sidera pini

## Additional information

### Use of AI

No use of AI was reported.

### Author ORCIDs

Wan-Ying Li https://orcid.org/0009-0006-7782-6670

Wen Jing https://orcid.org/0009-0009-8080-9910

Qian-Xin Guan https://orcid.org/0000-0002-7072-080X

Fang Wu https://orcid.org/0000-0002-1455-6486

### Data availability

All of the data that support the findings of this study are available in the main text or Supplementary Information.

## Supplementary materials

Supplementary material 1 Tree of *Coltricia* https://doi.org/10.3897/mycokeys.127.173104.suppl1 Data type nxs https://binary.pensoft.net/file/1512779This dataset is made available under the Open Database License (http://opendatacommons.org/licenses/odbl/1.0/). The Open Database License (ODbL) is a license agreement intended to allow users to freely share, modify, and use this Dataset while maintaining this same freedom for others, provided that the original source and author(s) are credited. Wan-Ying Li, Wen Jing, Qian-Xin Guan, Fang Wu

Supplementary material 2 Tree of *Sidera* https://doi.org/10.3897/mycokeys.127.173104.suppl2 Data type nxs https://binary.pensoft.net/file/1512780This dataset is made available under the Open Database License (http://opendatacommons.org/licenses/odbl/1.0/). The Open Database License (ODbL) is a license agreement intended to allow users to freely share, modify, and use this Dataset while maintaining this same freedom for others, provided that the original source and author(s) are credited. Wan-Ying Li, Wen Jing, Qian-Xin Guan, Fang Wu
